# The role of artificial intelligence in the treatment of obstructive sleep apnea

**DOI:** 10.1186/s40463-023-00621-0

**Published:** 2023-02-07

**Authors:** Hannah L. Brennan, Simon D. Kirby

**Affiliations:** Faculty of Medicine, Memorial University of Newfoundland and Labrador, 98 Pearltown Rd, St. John’s, NL A1G 1P3 Canada

**Keywords:** Obstructive sleep apnea, Treatment, Personalized treatment, Artificial intelligence, Machine learning

## Abstract

**Background:**

The first-line and most common treatment for obstructive sleep apnea is nasal continuous positive airway pressure, which serves as a pneumatic splint to stabilize the upper airway and is effective when used with appropriate adherence. Continuous positive airway pressure compliance rates remain significantly low despite machine improvements and compliance intervention. Other treatment options include oral appliances, myofunctional therapy, and surgery. The aim of this project is to elucidate the role of artificial intelligence within improving the treatment of obstructive sleep apnea.

**Methods:**

Related publications between 1999 and 2022 were reviewed from PubMed and Embase databases utilizing search terms “artificial intelligence,” “machine learning,” “obstructive sleep apnea,” and “treatment.” Both authors independently screened the results by title/abstract then by full text review. 126 non-duplicate articles were screened, 38 articles were included after title and abstract screen and 30 articles were included after full text review. The inclusion criteria are outline in the PICO framework and involved studies focused on artificial intelligence application in guiding and evaluating obstructive sleep apnea treatment. Non-English articles were excluded.

**Results:**

The role of artificial intelligence in the treatment of OSA was categorized into the following sections: Predicting treatment outcomes of various treatment options, Improving/Evaluating treatment, and Personalizing treatment with improving understanding of underlying mechanisms of OSA.

**Conclusions:**

Artificial intelligence has the capacity to improve the treatment of OSA through predicting outcomes of treatment options, evaluating the treatment the patient is currently utilizing and increasing understanding of the mechanisms that contribute to OSA disease process and physiology. Implementing AI in guiding treatment decisions allows patients to connect with treatment methods that would be most effective on an individual basis.

## Background

Obstructive sleep apnea (OSA) is a clinical condition characterized by a cessation of airflow due to upper airway obstruction with simultaneous respiratory effort present. In 2016 and 2017, 6.4% of adults in Canada reported they had been diagnosed with OSA [1]. Untreated OSA is associated with adverse health outcomes including cardiovascular disease, metabolic disease, cerebrovascular events, cognitive impairment and motor vehicle accidents [2]. While efficient diagnosis proses a barrier to treatment, the current treatment selection and pathway is also a major contributor to the disease process.

The first-line, gold standard treatment for OSA is nasal continuous positive airway pressure (CPAP), which serves as a pneumatic splint to stabilize the upper airway and is effective when used with appropriate adherence [3, 4]. However, adherence is a major challenge with CPAP treatment and is influenced by socio-demographic and psychosocial characteristics, disease severity and side effects [5]. Despite machine improvements and compliance interventions, CPAP non-adherence rates remain significantly high at 30–40% [6]. Other treatment options include behavioural modification, oral appliances (OAs), myofunctional therapy (MT), and surgical procedures.

Machine learning (ML), which is a subset of Artificial intelligence (AI), can be categorized into two broad groups: supervised and unsupervised learning. Unsupervised learning draws connections from data sets without labeled responses and have been used to identify different OSA subtypes. Supervised classification ML models requires labelled data sets to train the prediction models. Convolutional neural networks (CNN) are another ML method that requires minimal preprocessing and are well suited to analyze polysomnography (PSG) data. However one of the limitations is the larger data sets of information required to train these systems [7].

**Fig. 1:**
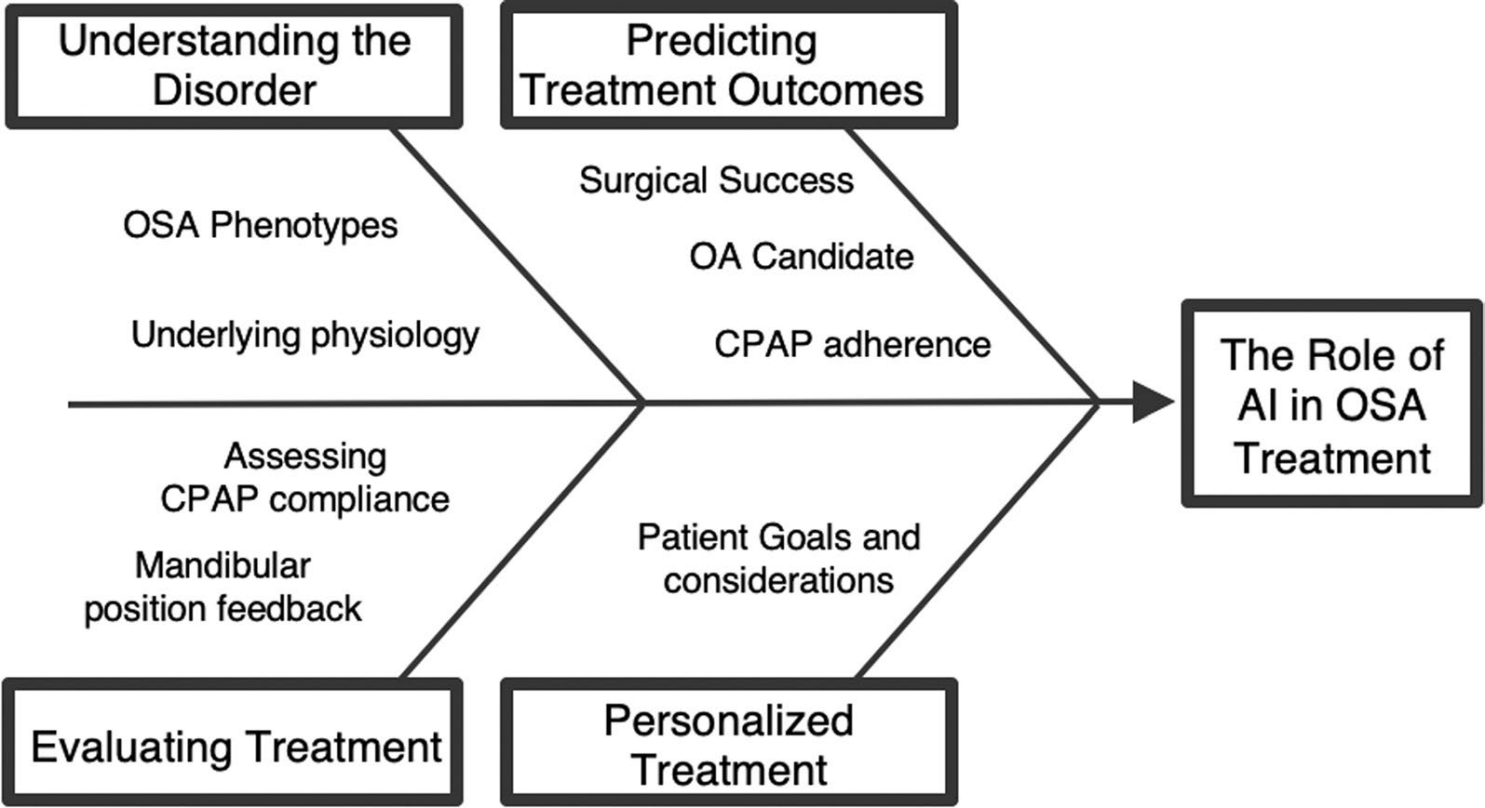
The role of AI in the treatment of OSA

There is a need to evaluate the role of AI in treatment of OSA, indicated by the significant non-compliance rate of CPAP, untreated OSA population and complexity of the disorder. The application of AI in the treatment of OSA can inform treatment selection, predict treatment success, evaluate current treatment and compliance and lead to personalized treatment approach. The role of AI in the treatment of OSA is outlined below and in Fig. 1.

## Methodology

Related publications between 1999 and 2022 were reviewed from PubMed and Embase databases utilizing search terms “artificial intelligence,” “machine learning,” “obstructive sleep apnea,” and “treatment.” Both authors independently screened the results by title/abstract then by full text review. 126 non-duplicate articles were screened, 38 articles were included after title and abstract screen and 30 articles were included after full text review. The inclusion criteria are outline in the PICO framework below and involved studies focused on artificial intelligence application in guiding and evaluating obstructive sleep apnea treatment. (P) The population focus of this study was adults with obstructive sleep apnea. (I) The intervention of interest was artificial intelligence in the context of treating obstructive sleep apnea. (C) The use of AI in treatment for OSA was mainly compared to the present treatment pathway and first line treatment of CPAP. (O) The role of AI in OSA treatment selection was identified in terms of improving/evaluating treatment and endotyping and clinical phenotyping to guide personalized management. Non-English articles were excluded from this review as well as articles that did not focused on AI’s implementation in OSA treatment.

## Main text

### Personalized medicine

OSA is a complex and heterogeneous disorder, varying in presenting symptoms, physiologic etiology, comorbidities and outcomes [8]. Therefore it is a disorder well suited for personalized approach to treatment, which can be designed to target the underlying OSA pathophysiology, including instability of ventilator control, insufficient dilator muscle response and low arousal threshold. Understanding the various phenotypes of OSA pathophysiology is important in personalizing treatment. Various studies have been conducted to predict arousal threshold, loop gain (consequent to hypersensitive ventilator control) and muscle compensation using PSG signals [9–11]. The framework of personalized or precision medicine involves prevention (risk prevention strategies), personalized (addressing individual phenotypes) and participation (patient involvement) [7]. This reflects a systematic approach to medicine supported by detailed biological data, analytical tools and linkage of patient outcome data. The rich data from PSG and AI analytic systems have the potential to advance the precision management of OSA [7].

Apnea–hypopnea index (AHI) remains the best studies metric of OSA severity, despite lacking the ability to fully reflect the complexity and genetic and biological underpinnings of the disorder [12]. A phenotyping approach can provide further insight into OSA severity and physiology and is an important step in personalizing medicine. Machine learning (ML) can be applied to identify phenotypes or previous unidentified patterns and complement the development of alternative metrics to quantify and describe OSA, rather than solely by AHI [12].

A study also employed supervised ML (decision tree learner) to develop a predictive algorithm for OSA endotypes from PSG and clinical data [13]. Such knowledge of the endotypes, upper-airway collapsibility, arousal threshold, loop gain and pharyngeal muscle responsiveness is essential to inform treatment avenues. Signal processing, control theory and ML have been proven effective for estimating the overall loop gain of the respiratory control system. Identification of mechanisms responsible for OSA would enable personalized treatment [14].

A study assessing the impact of OSA and treatment on the biological clock, revealed that ML approaches based on clock genes expression can distinguish between treated and un-treated OSA, and potentially play a role in treatment response monitoring [15]. Machine Learning (random forest model) has also been applied to kinematic driving data to detect lapses in treatment adherence and may be a valuable method for evaluating new treatments and adherence [16].

### CPAP treatment

A study by Zhu et al. identified genes in OSA and CPAP treatment by utilizing ML algorithms and a genetic database. This predictive model suggested individuals at high risk for OSA showed extensive activation of immune cells and pathways and higher expression of these genes which decreased after CPAP treatment. The information provided by ML in this setting can improve the identification of people with high risk of OSA as well as insight into CPAP treatment individual benefit [17]. Another study also used ML to identify biomarkers of the presence and severity of OSA, PAI-I, tPA and sE-Selectin, and demonstrated that they reduce with CPAP treatment and therefore may provide a measure of treatment response and guide preventative cardiovascular management for those patients identified as higher risk [18]. Similarly, ML and analysis of blood-based biomarkers can provide insight into the treatment of OSA and play a role in personalized management. A study highlighted 4 metabolites-signatures that provide an accuracy of 0.98 for OSA detection, and demonstrated a significant modulation of plasma metabolites previously altered by OSA following 6 months of CPAP therapy [19].

A study utilized ML to compare the compliance with CPAP therapy of patients with OSA at different points of treatment by building classifiers. The trial showed that month 3 was the time-point with the most accurate classifier of 84% in cross-validation and testing. Four variables (headaches, psychological symptoms, arterial hypertension and EuroQol visual analog scale) were reported relevant for prediction of CPAP compliance at each time point. Epworth and average nighttime hours were found the be important in prediction at months 1 and 3 [20].

Snore sound parameters have been utilized in studies with ML to classify the site of collapse of each hypopnea event into either lateral wall, palate and tongue base locations [21–23]. Using ML to increase the understanding of OSA physiology and etiology, particularly the location of upper airway collapse, can subsequently improve treatment selection and outcome. The ML approach with a model using linear discriminants to analyze audio signal was demonstrated to have fair accuracy in discriminating tongue and non-tongue collapse with overall accuracy of 81% and 64% accuracy for all sites of collapse classes [21]. Another study analyzing prediction of obstruction sites in OSA patients based on snoring sound parameters found the accuracies ranged from 60.4 to 92.2%. The study additionally concluded that snoring sound analysis does not seem to be a viable diagnostic modality for treatment selection [23].

The main challenge with CPAP treatment is poor adherence. ML methods have been developed to predict adherence, with a sensitivity of 68.6% and an area under the curve (AUC) of 72.9% [24]. Although the elucidation of factors that impact long-term CPAP adherence is complex, utilizing ML can allow for identification of patients with poor adherence, which can allow for further supports or other treatment selection. Additionally, ML has been used to help build a CPAP compliance-monitoring system to improve the management of OSA patients. Data from the CPAP device, including compliance, mask leaks and residual respiratory events was sent to the web database [25]. The intelligence monitoring system utilized this data to predicted expected compliance and provided feedback and interventions to increase compliance. The patients with this intervention had a mean of 1.14 h/day higher adjusted CPAP compliance than the control without the system. Overall this approach was cost effective and was associated with excellent patient satisfaction [25].

### Surgical treatment

The success of sleep surgery is highly variable with studies reporting a range from 45 to 78% in patients with OSA [26–28]. It is critical to establish a system for more accurate prediction of surgical success to avoid unnecessary surgeries and optimize the surgical treatment provided. A recent study by Kim et al. demonstrated the utility of ML models to predict surgical success, defined as postoperative AHI < 20 and a >/ = 50% reduction in preoperative AHI. The gradient boosting model (0.708) had significantly higher accuracy than logistic regression model (0.542) and the subjective prediction of a sleep surgeon (0.522) [29].

There are various surgical techniques to treat OSA, including uvulopalatal flap (UPF) technique which removes only the mucous membrane of the palate and uvula. Another option is the palatal muscle resection (PMR), where the levator veli palatine or palatoglossus muscle is cut and an anastomosis is performed. A study utilizing the several ML models, including logistic regression, tree-based models, support vector machine and neural networks, was conducted to determine the predicted success of UPF or PMR surgery. The ML models were developed with 15 variables including demography, PSG data, Friendman stage and Drug-induced sleep endoscopy (DISE) results and nasal or palate surgery. The study identified that the ML model with the highest accuracy was lasso logistic regression. The small sample size of 29 patients was a limiting factor in this study, and influenced which ML model was most accurate as the small data set compared to number of variables regularization methods are typically most useful [30].

Adenotonsillectomy (AT) is the main treatment approach for pediatric OSA. A study demonstrated how ML can be used to predict surgical candidates for pediatric OSA in order to help avoid unnecessary surgery in children with OSAS. An unsupervised ML technique, the K-means clustering method, was used to stratify patients into two groups depending on physiological and neurophysiological symptoms. The study demonstrated that children with mild symptoms could avoid AT and outlined an approach to more accurately predict who should be treated with AT [31]. Another study focused on pediatric OSA developed an algorithm to predict the need for AT and highlighted that tonsil size was the strongest predictor of AT [32]. ML has also be applied in the setting of deciding whether children need postoperative overnight monitoring following AT [33].

Hypoglossal nerve stimulator (HGNS) is a therapeutic option for moderate to severe OSA patients who are unable to tolerate CPAP. This treatment option requires surgical implantation and is associated with significant cost; therefore, it is critical to have a means to predict who will benefit from this option. Negative effort dependence (NED) is present when inspiratory airflow decreases despite increased driving pressure where certain patterns of NED are associated with different anatomic levels of upper airway collapse. A ML algorithm was developed to identify NED patterns in pre-treatment sleep studies and subsequently predict HGNS outcome. The ML algorithm accurately distinguished three NED patterns and found that the percentage of NED minimal breaths was significantly greater in responders than non-responders of HGNS [34].

Maxillomanibular advancement (MMA) and tracheostomies are also important surgical options to consider. MMA is a surgical treatment approach for OSA for patients who are unable to tolerate CPAP and have been refractory to other surgical modalities. MMA enlarges the airway by utilizing Le Fort I maxillary and sagittal split mandibular osteotomies [35]. This advancement of the maxilla and mandible draws the base of the tongue and soft palate forward reducing upper airway resistance. A meta-analysis of 45 studies, demonstrated the effectiveness of MMA as treatment for OSA with a significant reduction in AHI and respiratory disturbance index (RDI) post operatively, of 80.1% and 64.6% respectively [35]. Considering the invasive nature of the surgery and associated risks, including malocclusion, poor cosmetic result, facial numbness, jaw stiffness and postsurgical relapse of advancement, it is critical to optimize patient selection [35]. Similarly, Tracheostomy in the treatment of OSA is often explored once medical management fails or when the patient is not a candidate for other surgical treatment. A systematic review of 18 studies of OSA treated with tracheostomy, demonstrated significant decrease in AHI, desaturation index, day-time sleepiness and cardiovascular mortality [36]. However the effectiveness of this treatment option has to be balanced with the associated risks, implications of long term management of tracheostomy and impact of patient quality of life. There is a clear need for future studies focused on application of AI in informing the patient selection for both MMA and tracheostomies.

### Oral appliances

Four traits have been proposed to contribute to OSA, upper airway patency, impaired muscle responsiveness, low arousal threshold, and impaired ventilator control [37]. OA, also referred to as a mandibular advancement appliance device, passively and mechanically prevents closure of the upper airway by protruding the lower jaw and acts as a mandibular and tongue retainer. The health benefits, such as improved blood pressure and mortality are relatively equal between CPAP and OA over time, which may be due to greater compliance as OA tends to be used 6 h per night compared to 4–5 h for CPAP [38]. A major issue in oral appliance management of OSA is the lack of data on biological or demographic factors to predict treatment outcomes. Implementing AI in predicting treatment choices according to individual characteristics, can inform treatment selection [39]. Dutta et al. utilized developed and tested a ML-based model to predict OA therapy response according to standard AHI definitions, utilizing PSG variables, age, and body mass index (BMI). The ML model was trained with data from 45 individuals and subsequently tested on 17 participants. The trained model predicted OA therapy responders vs non-responders (AHI < 5events/h) had a mean accuracy of 91% using tenfold cross-validation. In the independent blinded validation, the model correctly predicted 100% of those patients who responded with AHI < 5events/h and 82% of those patients who had 50% reduction in AHI to < 20 events/h [40]. This study demonstrates the potential to harness routinely collected data and clinical data with ML-based approaches underpinned by OSA endotype concepts to help predict treatment outcomes [40].

A study by Mosca et al. investigated the ability to predict therapeutic success using a mandibular positioner with AI analytics. The AI system displayed had a high predictive accuracy. The predicted efficacious mandibular position was associated with therapeutic success in 83% of participants [41]. Another study was also conducted to assess the ability to prospectively identify therapeutic responders to oral appliance therapy with a feedback-controlled mandibular positioner. A ML classification system had a sensitivity and specificity of 85% and 93% respectively. The predicted mandibular protrusive position proved efficacious in 86% of cases. This ability to predict treatment outcomes using AI has the capacity to greatly impact patients route to effective treatment.

## Conclusion

Artificial intelligence has the capacity to improve the treatment of OSA through predicting outcomes of treatment options, evaluating the treatment the patient is currently utilizing and increasing understanding of the mechanisms that contribute to OSA disease process and physiology. Implementing AI in guiding treatment decisions allows patients to connect with treatment methods that would be most effective on an individual basis.

## Data Availability

Data sharing is not applicable to this article as no datasets were generated or analysed. All material included in the review was accessed through PubMed and outlined in the references section.

## Abbreviations

OSA: Obstructive sleep apnea
CPAP: Continuous positive airway pressure
OA: Oral appliances
MT: Myofunctional therapy
AI: Artificial intelligence
CNN: Convolutional neural networks
PSG: Polysomnography
AHI: Apnea–hypopnea index
ML: Machine learning
AUC: Area under the curve
UPF: Uvulopalatal flap
PMR: Palatal muscle resection
DISE: Drug-induced sleep endoscopy
AT: Adenotonsillectomy
HGNS: Hypoglossal nerve stimulator
NED: Negative effort dependence
MMA: Maxillomanibular advancement
RDI: Respiratory disturbance index
BMI: Body mass index

## References

[1] Gal J, Edjoc R. Sleep apnea in Canada, 2016 and 2017. Dly Stat Can. 2018;82-625-X.

[2] Jonas DE, Amick HR, Feltner C, Weber RP, Arvanitis M, Stine A (2017). Screening for obstructive sleep apnea in adults: evidence report and systematic review for the US preventive services task force. JAMA.

[3] Chang HP, Chen YF, Du JK (2020). Obstructive sleep apnea treatment in adults. Kaohsiung J Med Sci.

[4] Pavwoski P, Shelgikar AV (2017). Treatment options for obstructive sleep apnea. Neurol Clin Pract.

[5] Mehrtash M, Bakker JP, Ayas N (2019). Predictors of continuous positive airway pressure adherence in patients with obstructive sleep apnea. Lung.

[6] Rotenberg BW, Murariu D, Pang KP (2016). Trends in CPAP adherence over twenty years of data collection: a flattened curve. J Otolaryngol Head Neck Surg.

[7] de Chazal P, Sutherland K, Cistulli PA (2020). Advanced polysomnographic analysis for OSA: a pathway to personalized management?. Respirol Carlton Vic.

[8] Zinchuk AV, Gentry MJ, Concato J, Yaggi HK (2017). Phenotypes in obstructive sleep apnea: a definition, examples and evolution of approaches. Sleep Med Rev.

[9] Sands SA, Terrill PI, Edwards BA, Taranto Montemurro L, Azarbarzin A, Marques M (2018). Quantifying the arousal threshold using polysomnography in obstructive sleep apnea. Sleep.

[10] Terrill PI, Edwards BA, Nemati S, Butler JP, Owens RL, Eckert DJ (2015). Quantifying the ventilatory control contribution to sleep apnoea using polysomnography. Eur Respir J.

[11] Sands SA, Edwards BA, Terrill PI, Taranto-Montemurro L, Azarbarzin A, Marques M (2018). Phenotyping pharyngeal pathophysiology using polysomnography in patients with obstructive sleep apnea. Am J Respir Crit Care Med.

[12] Malhotra A, Ayappa I, Ayas N, Collop N, Kirsch D, Mcardle N (2021). Metrics of sleep apnea severity: beyond the apnea-hypopnea index. Sleep.

[13] Dutta R, Delaney G, Toson B, Jordan AS, White DP, Wellman A (2021). A novel model to estimate key obstructive sleep apnea endotypes from standard polysomnography and clinical data and their contribution to obstructive sleep apnea severity. Ann Am Thorac Soc.

[14] Nemati S, Orr J, Malhotra A (2014). Data-driven phenotyping. IEEE Pulse.

[15] Gaspar LS, Hesse J, Yalçin M, Santos B, Carvalhas-Almeida C, Ferreira M (2021). Long-term continuous positive airway pressure treatment ameliorates biological clock disruptions in obstructive sleep apnea. EBioMedicine.

[16] McDonald AD, Lee JD, Aksan NS, Dawson JD, Tippin J, Rizzo M (2017). Using kinematic driving data to detect sleep apnea treatment adherence. J Intell Transp Syst.

[17] Zhu J, Sanford LD, Ren R, Zhang Y, Tang X (2022). Multiple machine learning methods reveal key biomarkers of obstructive sleep apnea and continuous positive airway pressure treatment. Front Genet.

[18] Cederberg KLJ, Hanif U, Peris Sempere V, Hédou J, Leary EB, Schneider LD (2022). Proteomic biomarkers of the apnea hypopnea index and obstructive sleep apnea: insights into the pathophysiology of presence, severity, and treatment response. Int J Mol Sci.

[19] Pinilla L, Benítez ID, Santamaria-Martos F, Targa A, Moncusí-Moix A, Dalmases M (2022). Plasma profiling reveals a blood-based metabolic fingerprint of obstructive sleep apnea. Biomed Pharmacother.

[20] Rafael-Palou X, Turino C, Steblin A, Sánchez-de-la-Torre M, Barbé F, Vargiu E (2018). Comparative analysis of predictive methods for early assessment of compliance with continuous positive airway pressure therapy. BMC Med Inform Decis Mak.

[21] Sebastian A, Cistulli PA, Cohen G, de Chazal P (2021). Association of snoring characteristics with predominant site of collapse of upper airway in obstructive sleep apnea patients. Sleep.

[22] Sun J, Hu X, Chen C, Peng S, Ma Y (2020). Amplitude spectrum trend-based feature for excitation location classification from snore sounds. Physiol Meas.

[23] Huang Z, Aarab G, Ravesloot MJL, Zhou N, Bosschieter PFN, van Selms MKA (2021). Prediction of the obstruction sites in the upper airway in sleep-disordered breathing based on snoring sound parameters: a systematic review. Sleep Med.

[24] Scioscia G, Tondo P, Foschino Barbaro MP, Sabato R, Gallo C, Maci F (2022). Machine learning-based prediction of adherence to continuous positive airway pressure (CPAP) in obstructive sleep apnea (OSA). Inform Health Soc Care.

[25] Turino C, Benítez ID, Rafael-Palou X, Mayoral A, Lopera A, Pascual L (2021). Management and treatment of patients with obstructive sleep apnea using an intelligent monitoring system based on machine learning aiming to improve continuous positive airway pressure treatment compliance: randomized controlled trial. J Med Internet Res.

[26] Friedman M, Lin HC, Gurpinar B, Joseph NJ (2007). Minimally invasive single-stage multilevel treatment for obstructive sleep apnea/hypopnea syndrome. Laryngoscope.

[27] Richard W, Kox D, den Herder C, van Tinteren H, de Vries N (2007). One stage multilevel surgery (uvulopalatopharyngoplasty, hyoid suspension, radiofrequent ablation of the tongue base with/without genioglossus advancement), in obstructive sleep apnea syndrome. Eur Arch Oto-Rhino-Laryngol.

[28] Lin HC, Friedman M, Chang HW, Gurpinar B (2008). The efficacy of multilevel surgery of the upper airway in adults with obstructive sleep apnea/hypopnea syndrome. Laryngoscope.

[29] Kim JY, Kong HJ, Kim SH, Lee S, Kang SH, Han SC (2021). Machine learning-based preoperative datamining can predict the therapeutic outcome of sleep surgery in OSA subjects. Sci Rep.

[30] Yang SJ, Kim JS, Chung SK, Song YY (2021). Machine learning-based model for prediction of outcomes in palatal surgery for obstructive sleep apnoea. Clin Otolaryngol.

[31] Liu X, Pamula Y, Immanuel S, Kennedy D, Martin J, Baumert M (2022). Utilisation of machine learning to predict surgical candidates for the treatment of childhood upper airway obstruction. Sleep Breath Schlaf Atm.

[32] Heath DS, El-Hakim H, Al-Rahji Y, Eksteen E, Uwiera TC, Isaac A (2021). Development of a pediatric obstructive sleep apnea triage algorithm. J Otolaryngol Head Neck Surg.

[33] Bertoni D, Sterni LM, Pereira KD, Das G, Isaiah A (2020). Predicting polysomnographic severity thresholds in children using machine learning. Pediatr Res.

[34] Lou B, Rusk S, Nygate YN, Quintero L, Ishikawa O, Shikowitz M (2022). Association of hypoglossal nerve stimulator response with machine learning identified negative effort dependence patterns. Sleep Breath Schlaf Atm..

[35] Zaghi S, Holty JEC, Certal V, Abdullatif J, Guilleminault C, Powell NB (2016). Maxillomandibular advancement for treatment of obstructive sleep apnea: a meta-analysis. JAMA Otolaryngol Head Neck Surg.

[36] Camacho M, Certal V, Brietzke SE, Holty JEC, Guilleminault C, Capasso R (2014). Tracheostomy as treatment for adult obstructive sleep apnea: a systematic review and meta-analysis. Laryngoscope.

[37] Osman AM, Carter SG, Carberry JC, Eckert DJ (2018). Obstructive sleep apnea: current perspectives. Nat Sci Sleep.

[38] Vanderveken OM, Dieltjens M, Wouters K, De Backer WA, Van de Heyning PH, Braem MJ (2013). Objective measurement of compliance during oral appliance therapy for sleep-disordered breathing. Thorax.

[39] Lavigne GJ, Herrero Babiloni A, Beetz G, Dal Fabbro C, Sutherland K, Huynh N (2020). Critical issues in dental and medical management of obstructive sleep apnea. J Dent Res.

[40] Dutta R, Tong BK, Eckert DJ (2022). Development of a physiological-based model that uses standard polysomnography and clinical data to predict oral appliance treatment outcomes in obstructive sleep apnea. J Clin Sleep Med.

[41] Mosca EV, Bruehlmann S, Zouboules SM, Chiew AE, Westersund C, Hambrook DA (2022). In-home mandibular repositioning during sleep using MATRx plus predicts outcome and efficacious positioning for oral appliance treatment of obstructive sleep apnea. J Clin Sleep Med.

